# Co-existence of Multiple *Anaplasma* Species and Variants in Ticks Feeding on Hedgehogs or Cattle Poses Potential Threats of Anaplasmosis to Humans and Livestock in Eastern China

**DOI:** 10.3389/fmicb.2022.913650

**Published:** 2022-06-10

**Authors:** Yong Qi, Lele Ai, Changqiang Zhu, Yongfeng Lu, Ruichen Lv, Yingqing Mao, Nianhong Lu, Weilong Tan

**Affiliations:** ^1^Huadong Research Institute for Medicine and Biotechniques, Nanjing, China; ^2^Nanjing Bioengineering (Gene) Technology Center for Medicines, Nanjing, China; ^3^Institute of Rocket Force Medicine, State Key Laboratory of Trauma, Burns and Combined Injury, Army Medical University, Chongqing, China; ^4^Administration for Drug and Instrument Supervision and Inspection of PLAJLSF, Beijing, China

**Keywords:** *Anaplasma*, co-infection, co-existence, hedgehogs, ticks, *Erinaceus amurensis*

## Abstract

**Background:**

*Anaplasma* spp., causative agents of anaplasmosis, pose significant a threat to public health and economic losses in livestock farming. Co-infections/co-existence of various *Anaplasma* spp. may facilitate pathogen interactions and the emergence of novel variants, represent potential dangers to public health and economic losses from livestock farming, and raise challenges of detection and diagnosis. The information regarding co-infection/co-existence of *Anaplasma* in their vector ticks and wild animals is limited and needs urgent investigation.

**Methods:**

Wild hedgehogs and ticks from hedgehogs and cattle were collected from Jiangsu province, Eastern China, and DNA was extracted from hedgehog organs and tick homogenates. Various genera of species-specific polymerase chain reaction (PCR) or nested PCR amplifications targeting 16S ribosomal RNA (*rrs*), *msp4*, or *groEL* gene coupled with sequencing were conducted to identify *Anaplasma* spp.

**Results:**

*Anaplasma phagocytophilum* (1, 0.6%), *A. marginale* (2, 1.2%), *A. platys* variants xyn10pt-1 (13, 7.7%), xyn21pt-2 (3, 1.8%), and xyn3pt-3 (3, 1.8%), *A. bovis* variant cwp72bo-1 (12, 7.1%), and a novel *Candidatus* Cryptoplasma sp. (1, 0.6%) were identified in 168 *Haemaphysalis longicornis* ticks from cattle. *A. platys* variant xyn10pt-1 (20, 11.4%) and *A. bovis* variants cwp72bo-1 (12, 6.9%) and cwp55-36bo-2 (1, 0.6%) were detected in 173 *H. flava* ticks from hedgehogs. However, only *A. bovis* variant cwp72bo-1 (15, 46.7%) was identified in 32 *Erinaceus amurensis* hedgehogs. Various co-existence combinations were found only in ticks.

**Conclusion:**

The co-existence of various *Anaplasma* spp. and variants in *H. flava* and *H. longicornis* was detected for the first time in the world. The high infection rate of *A. bovis* in hedgehogs and its moderate infection rate in their parasitic ticks suggest that *Er. amurensis* hedgehog could be an important reservoir of *A. bovis*, rather than *A. platys*. Horizontal transmission of *Anaplasma* spp. may exist among different tick species *via* their shared hosts in the investigated area. This study provided epidemiological data that could be crucial for strategy development for early warning, prevention, and control of potential *Anaplasma* infections.

## Introduction

*Anaplasma* spp. are causative agents of anaplasmosis, with a significant impact on the health of a number of animals and human species, as well as economic losses in livestock farming systems (Battilani et al., 2017). Traditionally, the genus *Anaplasma* includes pathogenic *Anaplasma phagocytophilum, A. bovis, A. ovis, A. platys, A. marginale*, and *A. centrale*, in which the first four species can infect both humans and animals, while the remaining two are of veterinary importance, according to published evidence (Battilani et al., 2017; Lu et al., 2019). Tick-borne pathogens (TBPs) have attracted scholars' attention in molecular epidemiology, genetics, and pathobiology, leading to the discovery of novel species, such as *A. capra* and *A. odocoilei* (Tate et al., 2013; Li et al., 2015).

To date, ticks have been found to acquire various pathogenic species, such as parasites (*Babesia* spp.), bacteria (*Borrelia* spp., *Coxiella* spp., *Anaplasma* spp., *Ehrlichia* spp., and *Francisella* spp.), or viruses (tick-borne encephalitis virus and other tick-borne flaviviruses/phleboviruses), and multiple TBPs have been reported to co-exist within the same tick, which is not very surprising considering their great varieties (Cutler et al., 2021). Co-infections/co-existence of various TBPs may pose more potential health risks to humans and animals, raise the challenge of target pathogen detection and disease diagnosis, and facilitate pathogen interactions, resulting in potential recombination and novel mutant emergence. Previous studies aimed to provide valuable insights into co-infection (Cutler et al., 2021), and they mainly concentrated on the co-infection of TBPs with different genera. Co-infection of different *Anaplasma* spp. in domestic animals has been reported in several studies (Liu et al., 2012; Koh et al., 2018; Yang et al., 2020; Miranda et al., 2021), while there is a lack of information regarding *Anaplasma* co-infection/co-existence in their vector ticks and wild hedgehogs. Therefore, in the present study, we aimed to assess the prevalence and co-existence of various *Anaplasma* spp. or variants circulating in ticks feeding on cattle or wild hedgehogs, as well as their hedgehog hosts in Eastern China to provide epidemiological data to develop strategies for prevention and control of anaplasmosis.

## Materials and Methods

### Tick and Hedgehog Samples

Thirty-two hedgehogs were collected from several villages near Tieshan Temple (E 118°29' 6”, N 32°43' 55”) in Xuyi County, Jiangsu province, China, from May 2019 to October 2020. All of the hedgehog samples were road-killed or killed by domestic or stray dogs within 24 h of our obtaining them. After the collection of the ticks, the hedgehogs were immediately dissected for their hearts, livers, spleens, lungs, kidneys, brains, and intestines, and the specimens were stored at −80°C. In addition, ticks were also collected from 30 cattle from a farm located near the same sampling site.

In total, 341 ticks, including 173 adults from the hedgehogs and 168 adults from cattle, were collected and stored in tubes containing 70% ethanol. All the ticks were collected if the animal carried no more than 10 ticks; otherwise, 10 ticks were randomly collected from different parts of their bodies. The collected hedgehogs and ticks were first identified by their morphological features, as described previously (Feng, 1983), with an additional classification using a molecular method, as described below.

The Ethics Committee approved the animal experiments of the Huadong Research Institute for Medicine and Biotechniques. The animal care and treatment met the standards of the committee, with all efforts made to minimize the suffering of animals. Written informed consent was obtained from the cattle owners for the participation of their animals in the present study.

### DNA Purification

After twice washing with sterile phosphate-buffered saline (PBS), the ticks were individually homogenized in 1,000 μL of PBS using glass homogenizers. DNA from 200 μL of each tick homogenate or 10–30 mg of each hedgehog organ sample was extracted with a commercial DNeasy Blood & Tissue kit (Qiagen, Hilden, Germany), according to the manufacturer's instructions. The purified DNA was subsequently stored at −20°C before use.

### PCR Amplification and Sequencing

The Premix Ex Taq Version 2.0 kit (Takara, Beijing, China) was employed for the polymerase chain reaction (PCR) amplification, with 1 μL of the template and 1 μL of each primer (10 nM) used in each reaction. Molecular identification of each hedgehog or tick species was performed by amplifying the sequences of the mitochondrial 16S ribosomal RNA gene, as described previously (Sarri et al., 2014; Liu et al., 2016).

For screening *Anaplasma*-positive samples, a set of nested PCR primers (Table 1) targeting a short sequence of the 16S ribosomal RNA (*rrs*) gene with a length of about 280 bp was used, as previously described (Wen et al., 2003; Jiao et al., 2021). The amplified products were analyzed with 1.5% agarose gel electrophoresis and detected with GelStain Dye (Transgene, Beijing, China) under ultraviolet (UV) light. PCR products with expected sizes were excised from gels, extracted with a Gel Extraction kit (Sangon, Shanghai, China), and sequenced by Sangon Biotech Co., Ltd. (Shanghai, China). All the sequencing data were analyzed using SnapGene software (from Insightful Science; available at SnapGene.com). The obtained sequences were aligned using the BLAST search engine (https://blast.ncbi.nlm.nih.gov) to confirm the *Anaplasma*-positive samples.

**Table 1:** Primers used for PCR amplification in the present study.

**Target**	**Primer names**	**Nucleotide sequence (5^**′**^-3^**′**^)**	**Expected length (bp)**	**Annealing temperatures (**°**C)**
*Anaplasma* genus (*rrs* gene)	Eh-out1	TTGAGAGTTTGATCCTGGCTCAGAACG	660	55
	Eh-out2	CACCTCTACACTAGGAATTCCGCTATC		
	Eh-gs1	GTAATAACTGTATAATCCCTG	280	55
	Eh-gs2	GTACCGTCATTATCTTCCCTA		
*Anaplasma* spp. (*rrs* gene)	An16S1	GTCACTGACCCAACCTTAAATGGCTGC	1,432	51
	An16S2	ATCCTGGCTCAGAACGAACGCTGG		
	An16S3	GCGCCCTTCCGTTAAGAAGGATCTA	930	54
	An16S4	AGCTTAACACATGCAAGTCGAACGGA		
Four *Anaplasma* spp. (*rrs* gene, 1st)	AnU1F	AAGCTTAACACATGCAAGTCGAA	1,400	56
	AnU1R	AGTCACTGACCCAACCTTAAATG		
*A. phagocytophilum* (*rrs* gene, 2nd)	Anph2F	GTCGAACGGATTATTCTTTATAGCTTGC	926	56
	Anph2R	CCCTTCCGTTAAGAAGGATCTAATCTCC		
*A. platys* (*rrs* gene, 2nd)	Anpt2F	GATTTTTGTCGTAGCTTGCTATG	680	55
	Anpt2R	TAGCACTCATCGTTTACAGC		
*A. centrale* (*rrs* gene, 2nd)	Anct2F	CTGCTTTTAATACTGCAGGACTA	426	55
	Anct2R	ATGCAGCACCTGTGYGAGG		
*A. bovis* (*rrs* gene, 2nd)	Anbo2F	CTCGTAGCTTGCTATGAGAAC	551	55
	Anbo2R	TCTCCCGGACTCCAGTCTG		
*A. ovis* (*msp4* gene)	AnovMSP45	GGGAGCTCCTATGAATTACAGAGAATTGTTTAC	852	60
	AnovMSP43	CCGGATCCTTAGCTGAACAGGAATCTTGC		
*A. centrale* and *A. marginale* discrimination (*groEL* gene)	ACM1F	GCGCATTCTGGAGGCTG	1,479	55
	ACM1R	GACACAGCCAAGTCAAACGC		
	ACM2F	AATGAAGCGTGAAGTGGC	848	55
	ACM2R	GTACCACGCCTTCCTCAA		
Ticks (large subunit ribosomal RNA gene)	TickHF	GGTATTTTGACTATACAAAGGTATTG	278	54
	TickHR	TTATTACGCTGTTATCCCTAGAGTATT		

*First, the primer pair used in the first round for the amplification of the 16S rRNA gene shared by all the four Anaplasma spp., including A. phagocytophilum, A. platys, A. centrale, and A. bovis. Second, species-specific primer sets were used in the second round for the amplification of the 16S rRNA gene of the four Anaplasma spp*.

All the *Anaplasma*-positive samples were submitted for amplification for species identification using nested PCR targeting a longer sequence of the *rrs* gene about 930 bp as described previously (Barlough et al., 1996), with primers modified and optimized for the samples according to the former alignment results (Table 1). Moreover, to confirm the presence of multiple *Anaplasma* spp. in each sample, four different nested PCRs targeting the *rrs* gene of *A. phagocytophilum, A. platys, A. centrale*, and *A. bovis* were conducted for each sample, as previously described (Miranda et al., 2021). The same primer pairs were shared in the first round, and species-specific primer pairs were used in the second round (Table 1). In addition, the major surface protein 4 (*msp4*) gene was amplified to detect the presence of *A. ovis*, th and the *groEL* gene was amplified to distinguish *A. centrale* and *A. marginale*, as previously described with some modifications in primers (Table 1) (Byaruhanga et al., 2018; Miranda et al., 2021). In each PCR experiment, sterile distilled water and corresponding pathogen DNA samples were used as negative and positive control templates, respectively. The amplified products were sequenced on both strands by Sangon Biotech Co., Ltd. The PCR amplification for the samples to be sequenced was conducted in duplicate.

### Phylogenetic Analysis

The sequencing data were first analyzed using SnapGene software for quality evaluation and nucleotide sequence acquisition. The primer sequences were removed from both ends of the obtained sequences for subsequent alignment searches in GenBank using the BLAST search engine (https://blast.ncbi.nlm.nih.gov/Blast.cgi). Sequences, especially from available whole genomes in the GenBank database, with high homology, were selected for multiple sequence alignment using the ClustalW multiple alignment tool in MEGA 7.0 software. Phylogenetic analysis was conducted using MEGA 7.0 software, according to the maximum likelihood method with the Kimura two-parameter distance model and a bootstrap value of 1,000.

### Statistical Analysis

The positive rates of *Anaplasma* or the co-existence of various species/variants in different species of ticks feeding on hedgehogs or cattle were statistically analyzed using the Chi-square test, the continuity-adjusted Chi-square test, or Fisher's exact test according to the number of samples (n) and theoretical frequencies (T) using an online tool (available at http://quantpsy.org). *P* < 0.05 was considered statistically significant.

### Nucleotide Sequence Accession Numbers

The generated nucleotide sequences obtained in the present study were presented in Supplementary Table 1 and deposited into the GenBank database under accession numbers ranging from ON152887 to ON152896 and ON016525 to ON016529.

## Results

### Tick and Animal Taxonomic Classification

The hedgehogs were identified as *Erinaceus amurensis* by showing the highest nucleotide similarity (96.98%) of the amplified partial mitochondrial 16S ribosomal RNA gene sequences (Accession no. ON016529) with *Er. amurensis* in the GenBank database (Accession no. KX964606).

Similarly, the obtained mitochondrial 16S ribosomal RNA gene sequences from all the tick samples were aligned by the BLAST search engine, and tick species were identified. Among the 173 adult ticks collected from hedgehogs, 167 were determined to be *Haemaphysalis flava* (Accession nos. ON016525 to ON016528), and the other six were *H. longicornis* (Accession no. ON152895), while in the 168 ticks collected from cattle, one adult tick was identified to be *H. flava*, 15 adults were determined to be *Rhipicephalus microplus*, and the remaining 152 adults were *H. longicornis*. The predominant species of ticks harbored by hedgehogs and cattle were statistically different (χ2 = 295, *P* < 0.001).

### Identification of *Anaplasma* spp. or Variants

**Figure 1:**
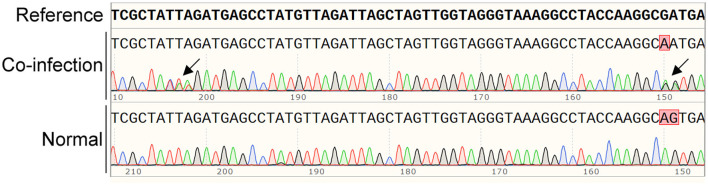
Typical co-infection with various *Anaplasma* spp. are indicated by overlapping in the sequencing analysis with SnapGene software. The overlapping peaks are indicated by black arrows.

The original sequencing data of each sample were analyzed with SnapGene software, and overlapping peaks were found in a few ticks (Figure 1), indicating the co-existence of various *Anaplsama* species in these samples. Thus, for each sample, various primer sets targeting the *rrs* gene were used to identify its containing *Anaplasma* spp. or variants.

**Figure 2:**
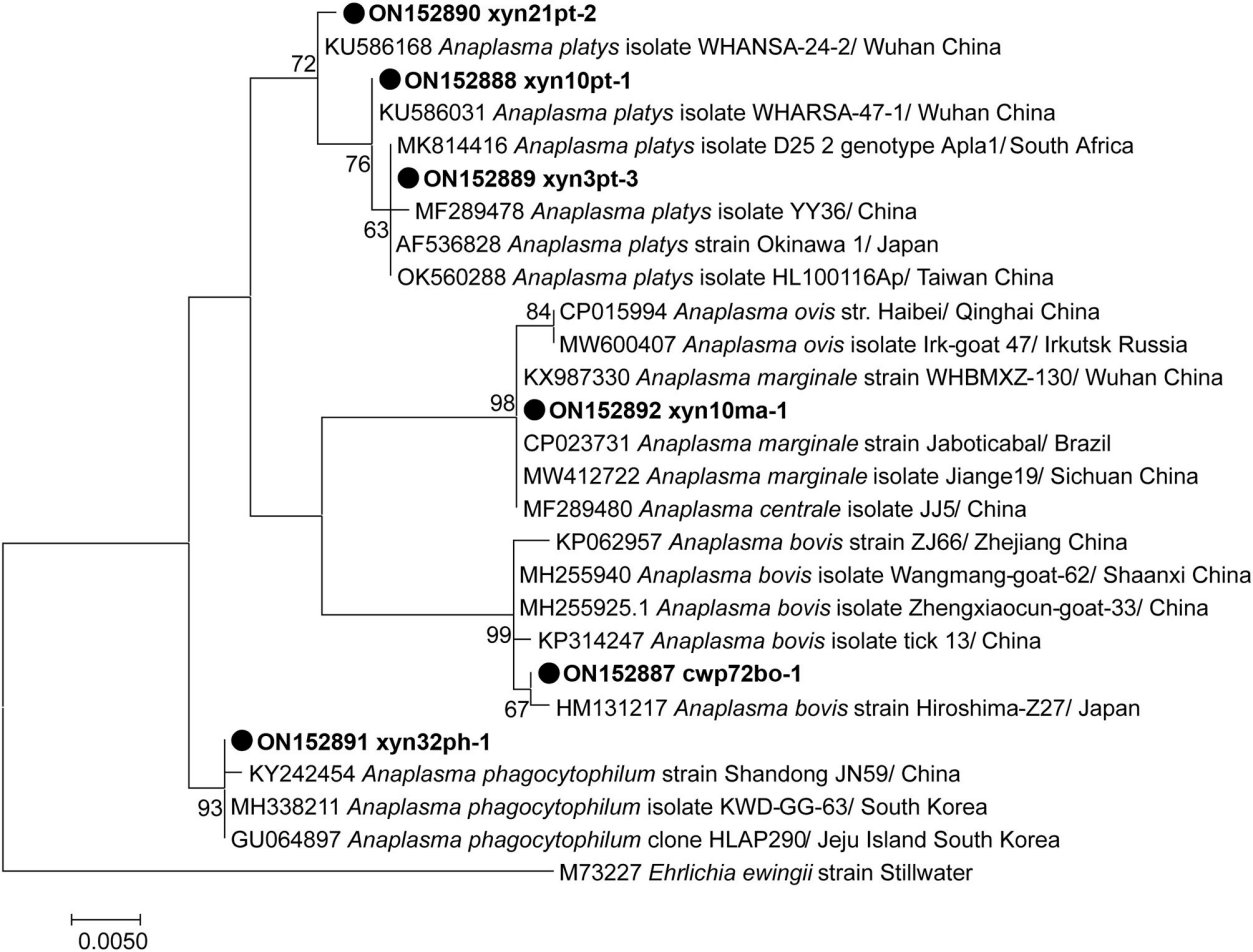
Phylogenetic tree of *Anaplasma* species based on partial *rrs* gene sequence. The sequences obtained in this study are indicated with black dots. The phylogenetic tree was generated using the maximum likelihood method with 1,000 replicates for bootstrap testing in MEGA 7.0 software. Only bootstrap values > 50% are shown. The scale bar indicates nucleotide substitutions per site. The species, locations, and GenBank accession numbers of the reference sequences are shown in each line.

Overall, 3, 2, 1, 1, and 1 representative partial *rrs* gene sequences of *A. platys* (Accession nos. ON152888 to ON152890), *A. bovis* (Accession nos. ON152887 and ON152893), *A. phagocytophilum* (Accession no. ON152891), *A. marginale* (Accession no. ON152892), and *Candidatus* Cryptoplasma sp. (Accession no. ON152894) were identified, respectively (Figure 2), of which six covered the similar position of the *rrs* gene and were phylogenetically analyzed together, as shown in Figure 2. The other two sequences, covering a relatively shorter part of *rrs*, were individually analyzed (Supplementary Figures 1, 2).

Analysis based on the partial 16S rRNA gene fragments revealed the presence of two distinct strains of *A. bovis*, namely variants cwp72bo-1 and cwp55-36bo-2, and three distinct strains of *A. platys*, including variants xyn10pt-1, xyn21pt-2, and xyn3pt-3. *A. bovis* variant cwp72bo-1 shared an identity rate of 100%, with the *A. bovis* (formerly named *Ehrlichia bovis*) sequence (Accession no. U03775) submitted by an institute from South Africa, while variant cwp55-36bo-2 (Supplementary Figure 1) shared an identity rate of 98.68% with this sequence. *A. platys* variants xyn10pt-1 and xyn21pt-2 showed the highest homology (identity rates of 100 and 99.87%, respectively) with the corresponding sequences (Accession nos. KU586031 and KU586168) identified in mosquitos in Wuhan, China, while the variant xyn3pt-3 sequence shared an identity rate of 100% with that of dozens of *A. platys* isolates or strains in the GenBank database. The identified *A. phagocytophilum* (xyn32ph-1 in Figure 1) sequence showed identity rates of 98.04–99.88% with those of various *A. phagocytophilum* strains or isolates in the GenBank database, indicating that it is a novel variant. The sequence xyn10ma-1 showed identity rates of 98.58–100% with *A. marginale* and 99.61–100% with its former subspecies, *A. centrale*. Therefore, a partial sequence of the *groEL* gene was amplified for further genotyping of this isolate. As a result, the sequence (Accession no. ON152896) shared an identity rate of 100% with various strains or isolates of *A. marginale*. The maximum identity rate was 98.89% with *A. centrale* strains or isolates, indicating that it belongs to *A. marginale*.

The short partial *rrs* gene sequence xyn113cr-1 revealed that this species was different from other *Anaplasma* spp. based on the highest identity rate of 97.08% with any variant of *Anaplasma* spp. (*A. phagocytophilum* strain Shandong JN59, Accession no. KY242454), while it showed a higher identity rate of 98.33% with 4 *Candidatus* Cryptoplasma sp. isolates (Accession nos. MG924904, KP276587, KP276585, and MW900167). The phylogenetic analysis (Supplementary Figure 2) showed that the sequence xyn113cr-1 and the *Candidatus* Cryptoplasma sp. isolates formed a separate clade on the phylogenetic tree with *Anaplasma* spp., which was distinct from the clade formed by *Ehrlichia* spp. and *Candidatus* Neoehrlichia spp., indicating that it belongs to the novel genus described previously by Eshoo et al. (2015). However, it was not clustered with these *Candidatus* Cryptoplasma sp. isolates (bootstrap support of 97%, Supplementary Figure 2), and the divergence between the sequence xyn113cr-1 and these *Candidatus* Cryptoplasma sp. isolates was greater than most of the variations between species within the genus *Anaplasma, Ehrlichia*, or ‘*Candidatus* Neoehrlichia’, thereby providing strong phylogenetic evidence for the recognition of a novel *Candidatus* Cryptoplasma sp.

### Prevalence of *Anaplasma* spp. or Variants in Individual Ticks and Hedgehogs

The presence of *Anaplasma* spp. in each sample was detected by nested PCR combined with sequencing. As a result, 15.0% (26/173) of ticks from hedgehogs and 7.7% (13/168) of ticks from cattle were positive, with a statistically significant difference (χ2=4.3, *P*<0.05). In the present study, all the *Anaplasma*-positive ticks from hedgehogs were *H. flava*, and those from cattle were *H. longicornis*. In contrast, the differences in positive rates between *H. flava* and *H. longicornis* from hedgehogs or between *H. longicornis* and non-*H. longicornis* from cattle were not statistically significant (*P* > 0.05). In hedgehogs, 43.8% (14/32) were *Anaplasma*-positive, which was significantly higher than that in their parasitic ticks (χ2 = 14.2, *P* < 0.001). Different positive rates were found in different organs. Briefly, *Anaplasma* spp. was detected in 0% (0/32) of the brain samples, 25% (8/32) of the spleen samples, 25% (8/32) of the lung samples, 18.8% (6/32) of the liver samples, 15.6% (5/32) of the heart samples, 12.5% (4/32) of the intestine samples, and 9.4% (3/32) of the kidney samples.

The predominant *Anaplasma* spp. variants were *A. bovis* variant cwp72bo-1 (both in ticks and hedgehogs) and *A. platys* variant xyn10pt-1 (in ticks). In general, the *A. bovis* variant cwp72bo-1-positive samples accounted for 100% (14/14), 76.9% (20/26), and 92.3% (12/13) of the total *Anaplasma*-positive hedgehogs, ticks feeding on hedgehogs, and ticks feeding on cattle, respectively; the *A. platys* variant xyn10pt-1-positive samples accounted for 46.2% (12/26) and 100% (13/13) of the total *Anaplasma*-positive ticks feeding on hedgehogs and cattle, respectively. The *A. bovis* variant cwp55-36bo-2 was only found in one tick from hedgehogs, while *A. platys* variants xyn21pt-2 and xyn3pt-3, *A. marginale, A. phagocytophilum*, and *Candidatus* Cryptoplasma sp., were found in 3, 3, 4, 1, and 1 tick from cattle, respectively.

### Co-existence of *Anaplasma* spp. or Variants in Individual Ticks and Hedgehogs

In hedgehogs, co-infection/co-existence of various *Anaplasma* spp. or variants was not observed, and only *A. bovis* variant cwp72bo-1 was identified (Table 2). However, in the hedgehog-attached ticks, *A. platys* variant xyn10pt-1 and the 2 *A. bovis* variants were detected, in which co-existence of these 3 *Anaplasma* spp. or variants was found in one tick, and co-existence of *A. platys* variant xyn10pt-1 and *A. bovis* variant cwp72bo-1 was observed in five ticks.

**Table 2:** Co-existence/co-infections of various *Anaplasma* spp. or variants in various samples.

***Anaplasma*** **spp. or variants**	**Number of individuals positive for single** **and co-infections/co-existence**		
	**Ticks from hedgehogs (*n* = 175)**	**Hedgehogs (*n* = 30)**	**Ticks from cattle** **(*n* = 168)**
Quintuple	0	0	1
*A. bovis* cwp72bo-1, *A. platys* xyn10pt-1, *A. platys* xyn21pt-2, *A. marginale*, and *A. phagocytophilum*	0	0	1
Quadruple	0	0	2
*A. bovis* cwp72bo-1, *A. platys* xyn10pt-1, *A. platys* xyn21pt-2, and *A. platys* xyn3pt-3	0	0	2
Triple	1	0	5
*A. bovis* cwp72bo-1, *A. platys* xyn10pt-1, and *A. marginale*	0	0	3
*A. bovis* cwp72bo-1, *A. platys* xyn10pt-1, and *A. platys* xyn3pt-3	0	0	1
*A. bovis* cwp72bo-1, *A. platys* xyn10pt-1, and *Candidatus*	0	0	1
Cryptoplasma sp.			
*A. bovis* cwp72bo-1, *A. bovis* cwp55-36bo-2, and *A. platys* xyn10pt-1	1	0	0
Double	5	0	4
*A. bovis* cwp72bo-1, and *A. platys* xyn10pt-1,	5	0	4
Single	20	0	1
*A. platys* xyn10pt-1	6	0	1
*A. bovis* cwp72bo-1	14	14	0
None	149	16	155

In ticks from cattle, the co-existence of various *Anaplasma* spp. or variants was more complicated. As indicated in Table 2, single existence or quintuple co-existence of *Anaplasma* spp. or variants was rarely observed (in one tick individually), while triple and dual-existences were frequently detected (in five and four ticks, respectively). Surprisingly, the co-existence of up to three different *A. platys* variants in one tick was also observed.

In all the ticks with co-existence, whether from hedgehogs or cattle, *A. bovis* variant cwp72bo-1 and *A. platys* variant xyn10pt-1 were identified. In *Anaplasma*-positive samples, significantly more co-existence was found in ticks from cattle than in those from hedgehogs (χ2 = 14.0, *P* < 0.001).

## Discussion

**Figure 3:**
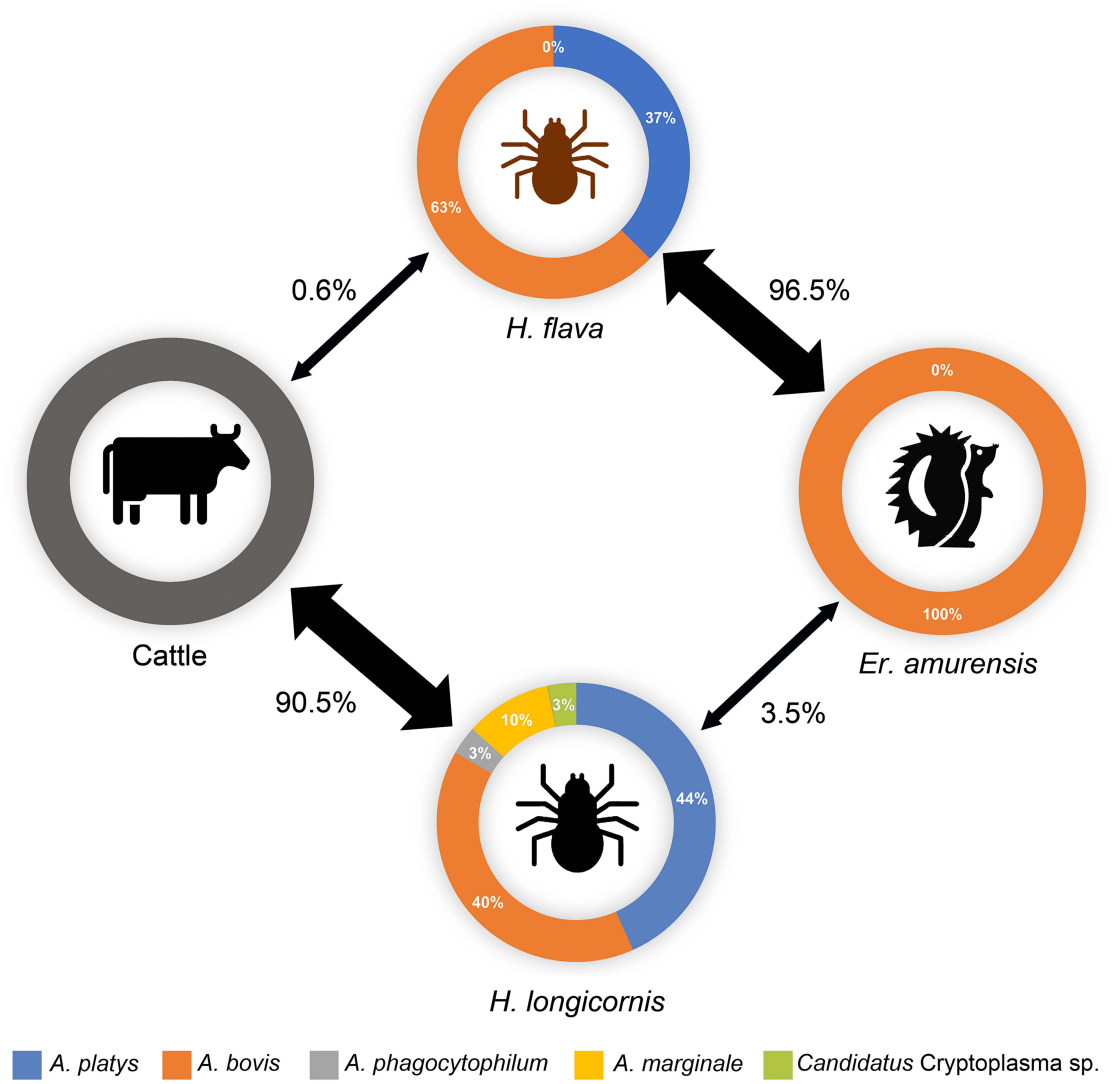
Potential transmission circle of *Anaplasma* spp. in ticks and animals in the investigated area. The thickness of the arrow represents the parasitic rates (number of ticks of specific species/number of total parasitic ticks) of each tick species on hedgehog or cattle hosts, which are also indicated beside the arrows. The relative abundances of each *Anaplasma* spp. in positive samples are indicated. Data on cattle were not obtained.

In this study, the main parasitic ticks of hedgehogs and cattle were *H. flava* and *H. longicornis*, respectively, while only 0.6% of the cattle-attached ticks were *H. flava*, and 3.5% of the hedgehog-attached ticks were *H. longicornis* (Figure 3). The predominance of *H. flava* in hedgehogs was also observed in Central China, where all the 125 ticks collected from hedgehogs were *H. flava* (Fang et al., 2021). Several genera of ticks have their own set of preferred hosts, and the associations of ticks with their hosts indicate their roles in TBP circulation (Islam et al., 2006; Spengler and Estrada-Peña, 2018). Therefore, to some extent, the acquisition of epidemiological information on hosts and their predominantly parasitic tick species, as well as the fact that TBPs that harbor and transmit, may be advantageous for the development of prevention and control strategies for tick-borne diseases (Chisu et al., 2020; Song et al., 2021).

With increasing awareness about TBPs, the co-infections/co-existence of multiple pathogens in ticks or their hosts have greatly attracted scholars' attention (Cutler et al., 2021; Fang et al., 2021; Jiao et al., 2021; Soong and Dong, 2021). *Anaplasma* spp. are tick-transmitted obligate intracellular bacteria that may infect numerous wild and domestic animals and humans through tick biting. Hedgehogs have been suggested to be an appropriate reservoir for some *Anaplasma* spp. (Silaghi et al., 2012; Jahfari et al., 2017; Khodadadi et al., 2021), and their harboring TBPs are more easily transmitted to domestic animals and humans (Chisu et al., 2020). On the one hand, the activity area of hedgehogs for searching for food and shelter typically intersects with that of humans in (sub) urban areas, such as parks, gardens, urban green areas, farmlands, livestock farms, etc.; on the other hand, people mainly take hedgehogs into their homes as pets or to rescue injured ones. These possible contacts with hedgehogs or their contaminated environment will expose people to various zoonotic pathogens that hedgehogs harbor (Ruszkowski et al., 2021). Hence, it seems equally important to investigate the prevalence and co-infections/co-existence of *Anaplasma* spp. in wild hedgehogs and ticks to assess their transmission risks. Several spirochete species have been reported to co-exist in ticks (Herrmann et al., 2013), while co-existence/co-infections of various *Anaplasma* spp. or variants have only been reported in domestic animals (e.g., goats, sheep, cattle, and dogs), rather than in ticks or wild hedgehogs (Liu et al., 2012; Yang et al., 2015, 2020; Seo et al., 2018; Miranda et al., 2021). To our knowledge, this is the first study that investigated the co-infections/co-existence of various *Anaplasma* spp. or variants in wild hedgehogs and ticks and confirmed their co-existence in *H. flava* and *H. longicornis* ticks.

Although significantly varied in species, ticks from hedgehogs or cattle, *Anaplasma* spp. prevalence or co-infection/co-existence rates were always found to contain *A. bovis* variant cwp72bo-1 coupled with *A. platys* variant xyn10pt-1 in the co-existence. These two variants were both predominant in the ticks from hedgehogs or cattle, indicating that they are the main endemic *Anaplasma* spp. variants in this region, and there may be a horizontal transmission of *Anaplasma* spp. between *H. flava* and *H. longicornis via* their shared hosts (Figure 3).

*A. bovis* was detected in *Er. amurensis* hedgehogs with a high positive rate (43.8%), while *A. platys* was found in none of the hedgehogs in the present study. *A. bovis* was reported to infect small mammals and ruminants monocytes and cause anaplasmosis (Liu et al., 2012; Ben Said et al., 2018), while *A. platys*, as the etiological agent of the infectious canine cyclic thrombocytopenia by infecting the platelets of dogs, can cause human infections (Harvey et al., 1978; Maggi et al., 2013; Arraga-Alvarado et al., 2014). To our knowledge, no previous study has reported that *A. platys* could infect hedgehogs. Thus, *Er. amurensis* may be an important reservoir for certain *Anaplasma* spp., like *A. bovis* in the present study and *A. phagocytophilum* in the previous study (Silaghi et al., 2012), rather than *A. platys*. However, more hedgehogs should be further investigated, considering the small size of animals included in the present study. In the infected hedgehogs, *Anaplasma*-positive or negative might be found in the same organs from different individuals, resulting in different positive rates in different organs. This phenomenon, which was also observed in a sheep model (Almazán et al., 2020), may be due to individual variations, including their immune status, pathogen loads, and co-existence of other bacteria. However, in general, higher rates were observed in the spleens, livers, and lungs of the hedgehogs in the present study, which is consistent with results obtained from another model using C3H/HeJ mice (Blas-Machado et al., 2007), in which scholars found splenomegaly and microscopical lesions in the lungs, spleens, and livers, rather than in other organs or tissues of the *A. phagocytophilum*-infected mice.

The co-existence of various *Anaplasma* spp. or variants in ticks may be due to transstadial transmission or their blood meal from the present host. For instance, in the present study, the *A. bovis* carried by *H. flava* could have come from tick biting hedgehogs or from transstadial transmission, while *A. platys* most likely came from transstadial transmission. Nevertheless, a previous study reported that hosts harboring diverse *A. phagocytophilum* strains may enable the emergence of new variants or types *via* bacterial recombination (Tegtmeyer et al., 2019), indicating that the co-existence of various *Anaplasma* spp. or variants in the present study has the potential to lead to the emergence of novel variants and cause pathogenic risks in the investigated area. The observations of various variants of *A. platys* or *A. bovis* in this area, and these minority variants (*A. bovis* variant cwp55-36bo-2 or *A. platys* variants xyn21pt-2 and xyn3pt-3) were always detected together in ticks containing their corresponding majority variants (*A. bovis* variant cwp72bo-1 or *A. platys* variant xyn10pt-1), supporting the hypothesis mentioned above.

*A. phagocytophilum* and *A. marginale* are the most significant disease-causing pathogens in the genus *Anaplasma* (Battilani et al., 2017). *A. phagocytophilum*, the agent of human granulocytic anaplasmosis (HGA), also causes tick-borne fever in sheep and goats and pasture fever in cattle (Battilani et al., 2017). *A. marginale* causes bovine anaplasmosis, a crucial rickettsial disease in cattle worldwide, which is characterized by severe anemia, abortions, loss of weight and milk production, and high morbidity and mortality (Quiroz-Castañeda et al., 2016). Both agents were detected in ticks feeding on cattle, indicating their potential threat to both humans and livestock in this area. However, the prevalence of these pathogens in humans or livestock needs further investigation.

Moreover, a novel *Candidatus* Cryptoplasma sp. was also identified in a tick from cattle. Similar sequences were found in China and South Korea (Accession nos. JN715833, KT596737, and GU075699-GU075704) and are classified as uncultured *Anaplasma* spp. Compared with *Anaplasma* spp., the sequences shared higher identity rates than those from *Candidatus* Cryptoplasma spp., which were recently characterized and named in 2015 (Eshoo et al., 2015). However, whether this bacterium could cause human infections requires further investigation.

Several studies have investigated the prevalence of *Anaplasma* spp. in animal and tick hosts in the Northeastern, Northwestern, Southern, and Central China (Jiang et al., 2011; Liu et al., 2012; Zhang et al., 2012; Li et al., 2015; Yang et al., 2015; Wei et al., 2016; Fang et al., 2021; Jiao et al., 2021; Yan et al., 2021), while few studies have been carried out in Eastern China (Lu et al., 2019; Yang et al., 2020). The *Anaplasma* species, their distribution, and prevalence in Eastern China have remained elusive. The present study filled the gaps by providing useful epidemiological data related to the pathogen species and their presence in the ticks or animal hosts in Jiangsu province, Eastern China.

Some limitations exist in the present study. First, although various PCR amplifications toward the *rrs* gene were used to detect different *Anaplasma* spp., there is still the possibility of missed detection of other possible *Anaplasma* spp. or variants due to the diversity of pathogens in the samples. Second, the detection limit of the typically used nested PCR may influence the positive rates, and a more sensitive real-time PCR method may overcome the bias. Third, the co-infections/co-existence make it potentially difficult to achieve precise detection or typing of *Anaplasma* spp. or variants. For instance, when a sample contains several variants with only a few nucleotide distinctions in their conserved genes (e.g., *rrs* or *groEL*), it will be challenging to indicate which *rrs* and *groEL* genes sequenced belong to the same strain. Hence, for further characterization, pathogen isolation should be conducted. Finally, the co-infection agents in the cattle remain to be defined.

In conclusion, we investigated the co-infections/co-existence of various *Anaplasma* spp. and variants in hedgehogs and ticks feeding on hedgehogs and cattle for the first time. Important pathogenic *Anaplasma* spp., including *A. phagocytophilum, A. marginale*, 3 *A. platys* variants, and 2 *A. bovis* variants, as well as a novel *Candidatus* Cryptoplasma sp. were identified, with various prevalence rates in ticks and hedgehogs, and different co-existence combinations were observed only in ticks, rather than in hedgehogs. Hedgehogs may be important reservoirs for *A. bovis*, rather than for *A. platys*. Horizontal transmission of *Anaplasma* spp. may exist between different tick species *via* their shared hosts. Co-existence of various *Anaplasma* spp. or variants in the present study may facilitate the emergence of novel variants, pose potential threats to public health as well as economic losses from livestock farming, and raise challenges for detection and diagnosis in the investigated area. The present study, with the identification of important pathogenic *Anaplasma* spp. and their co-infections/co-existence in the ticks, provided epidemiological data that could be crucial for strategy development in early warning, prevention, and control of potential *Anaplasma* infections.

## Data Availability Statement

The data presented in the study are deposited in the GenBank database, accession numbers ON152887 to ON152896 and ON016525 to ON016529.

## Ethics Statement

The animal study was reviewed and approved by Ethics Committee of Huadong Research Institute for Medicine and Biotechniques. Written informed consent was obtained from the owners for the participation of their animals in this study.

## Author Contributions

YQ, LA, and WT conceptualized the study. YQ and LA curated the data and formal analysis was performed by CZ and YM. WT and YQ provided the funding. The methodology was designed by RL and NL. The software was designed by YL. The original draft was prepared by YQ, while WT and YQ were involved in writing, reviewing, and editing. All authors contributed to the article and approved the submitted version.

## Funding

This work was supported by Medical Science and Technology Projects (19SWAQ04 and 2020QN06357), a Jiangsu Social Development Project (BE2017620), and the National Natural Science Foundation of China (U1602223).

## Conflict of Interest

The authors declare that the research was conducted in the absence of any commercial or financial relationships that could be construed as a potential conflict of interest.

## Publisher's Note

All claims expressed in this article are solely those of the authors and do not necessarily represent those of their affiliated organizations, or those of the publisher, the editors and the reviewers. Any product that may be evaluated in this article, or claim that may be made by its manufacturer, is not guaranteed or endorsed by the publisher.
