# Supplementary material for: Co-existence of Multiple Anaplasma Species and Variants in Ticks Feeding on Hedgehogs or Cattle Poses Potential Threats of Anaplasmosis to Humans and Livestock in Eastern China
Source: Front Microbiol. 2022 Jun 10;13:913650. doi: 10.3389/fmicb.2022.913650 (PMC9226643; doi:10.3389/fmicb.2022.913650)
Supplement: Supplementary file 1 [file Table_1.docx]

**Supplementary Table 1 Accession Nos. and sequences obtained in the study.**

| Accession No. | Name | Sequence (5’-3’) |
| --- | --- | --- |
| ON152887 | cwp72bo-1 | TTGTTCTCGTAGCTTGCTATGAGAACAATTAGTGGCAGACGGGTGAGTAATGCATAGGAATCTACCTAGTAGTATAGGATAGCCACTAGAAGTGGTGGGTAATACTGTATAATCCCTGCGGGGGAAAGATTTATCGCTACATGATGAGCCTATGTTAGATTAGCTAGTTGGTGGGGTAATGGCCTACCAAGGCAGTGATCTATAGCTGGTCTGAGAGGATGATCAGCCACACTGGAACTGAGACACGGTCCAGACTCCTACGGGAGGCAGCAGTGGGGAATATTGGACAATGGGCGCAAGCCTGATCCAGCTATGCCGCGTGAGTGAGGAAGGCCTTAGGGTTGTAAAACTCTTTCAGTGGGGAAGATAATGACGGTACCCACAGAAGAAGTCCCGGCAAACTCCGTGCCAGCAGCCGCGGTAATACGGAGGGGGCAAGCGTTGTTCGGAATTATTGGGCGTAAAGGGCATGTAGGTGGTTTGGTTAGTTAAAGGTGAAATGCCAGGGCTTAACCCTGGAGCTGCTTTTAATACTGCCAGACTGGAGTCCGGGAGAGGATAGCGGAATTCCTAGTGTAGAGGTGAAATTCGTAGATATTAGGAGGAACACCAGTGGCGAAGGCGGCTATCTGGTCCGGTACTGACACTGAGGTGCGAAAGCGTGGGGAGCAAACAGGATTAGATACCCTGGTAGTCCACGCTGTAAACGATGAGTGCTGGATGTGGGGGTTTTTACCTCCGTGTTGTAGCTAACGCGTTAAGCACTCCGCCTGGGGACTACGGTCGCAAGACTAAAACTCAAAGGAATTGACGGGGACCCGCACAAGCGGTGGAGCATGTGGTTTAATTCGATGCAACGCGAAAAACCTTACCACTCCTTGACATGAAGAT |
| ON152888 | xyn10pt-1 | TTTTTATCATAGCTTGCTATGATAAAAATTAGTGGCAGACGGGTGAGTAATGCATAGGAATCTACCTAGTAGTATGGGATAGCCACTAGAAATGGTGGGTAATACTGTATAATCCCTGCGGGGGAAAGATTTATCGCTATTAGATGAGCCTATGTTAGATTAGCTAGTTGGTAGGGTAAAGGCCTACCAAGGCAGTGATCTATAGCTGGTCTGAGAGGATGATCAGCCACACTGGAACTGAGATACGGTCCAGACTCCTACGGGAGGCAGCAGTGGGGAATATTGGACAATGGGCGCAAGCCTGATCCAGCTATGCCGCGTGAGTGAGGAAGGCCTTAGGGTTGTAAAACTCTTTCAGTGGGGAAGATAATGACGGTACCCACAGAAGAAGTCCCGGCAAACTCCGTGCCAGCAGCCGCGGTAATACGGAGGGGGCAAGCGTTGTTCGGAATTATTGGGCGTAAAGGGCATGTAGGCGGTTCGGTAAGTTAAAGGTGAAATGCCAGGGCTTAACCCTGGAGCTGCTTTTAATACTGCCAGACTCGAGTCCGGGAGAGGATAGCGGAATTCCTAGTGTAGAGGTGAAATTCGTAGATATTAGGAGGAACACCAGTGGCGAAGGCGGCTATCTGGTCCGGTACTGACGCTGAGGTGCGAAAGCGTGGGGAGCAAACAGGATTAGATACCCTGGTAGTCCACGCTGTAAACGATGAGTGCTGAATGTGGGGGCGTTTTGTCTCTGTGTTGTAGCTAACGCGTTAAGCACTCCGCCTGGGGACTACGGTCGCAAGACTAAAACTCAAAGGAATTGACGGGGACCCGCACAAGCGGTGGAGCATGTGGTTTAATTCGATGCAACGCGAAGAACCTTACCACTTCTTGACATGGAGAT |
| ON152889 | xyn3pt-3 | TATGATAAAAATTAGTGGCAGACGGGTGAGTAATGCATAGGAATCTACCTAGTAGTATGGGATAGCCACTAGAAATGGTGGGTAATACTGTATAATCCCTGCGGGGGAAAGATTTATCGCTATTAGATGAGCCTATGTTAGATTAGCTAGTTGGTAGGGTAAAGGCCTACCAAGGCAGTGATCTATAGCTGGTCTGAGAGGATGATCAGCCACACTGGAACTGAGATACGGTCCAGACTCCTACGGGAGGCAGCAGTGGGGAATATTGGACAATGGGCGCAAGCCTGATCCAGCTATGCCGCGTGAGTGAGGAAGGCCTTAGGGTTGTAAAACTCTTTCAGTGGGGAAGATAATGACGGTACCCACAGAAGAAGTCCCGGCAAACTCCGTGCCAGCAGCCGCGGTAATACGGAGGGGGCAAGCGTTGTTCGGAATTATTGGGCGTAAAGGGCATGTAGGCGGTTCGGTAAGTTAAAGGTGAAATGCCAGGGCTTAACCCTGGAGCTGCTTTTAATACTGCCAGACTCGAGTCCGGGAGAGGATAGCGGAATTCCTAGTGTAGAGGTGAAATTCGTAGATATTAGGAGGAACACCAGTGGCGAAGGCGGCTATCTGGTCCGGTACTGACGCTGAGGTGCGAAAGCGTGGGGAGCAAACAGGATTAGATACCCTGGTAGTCCACGCTGTAAACGATGAGTGCTGAATGTGGGGACGTTTTGT |
| ON152890 | xyn21pt-2 | CGGGAGAAAGATTTATCGCTATTAGATGAGCCTATGTTAGATTAGCTAGTTGGTAGGGTAAAGGCCTACCAAGGCAGTGATCTATAGCTGGTCTGAGAGGATGATCAGCCACACTGGAACTGAGATACGGTCCAGACTCCTACGGGAGGCAGCAGTGGGGAATATTGGACAATGGGCGCAAGCCTGATCCAGCTATGCCGCGTGAGTGAGGAAGGCCTTAGGGTTGTAAAACTCTTTCAGTGGGGAAGATAATGACGGTACCCACAGAAGAAGTCCCGGCAAACTCCGTGCCAGCAGCCGCGGTAATACGGAGGGGGCAAGCGTTGTTCGGAATTATTGGGCGTAAAGGGCATGTAGGCGGTTCGGTAAGTTAAAGGTGAAATGCCAGGGCTTAACCCTGGAGCTGCTTTTAATACTGCCAGACTAGAGACCGGGAGAGGATAGCGGAATTCCTAGTGTAGAGGTGAAATTCGTAGATATTAGGAGGAACACCAGTGGCGAAGGCGGCTATCTGGTCCGGTACTGACGCTGAGGTGCGAAAGCGTGGGGAGCAAACAGGATTAGATACCCTGGTAGTCCACGCTGTAAACGATGAGTGCTGAATGTGGGGGTGTTTGCCTCTGTGTTGTAGCTAACGCGTTAAGCACTCCGCCTGGGGACTACGGTCGCAAGACTAAAACTCAAAGGAATTGACGGGGACCCGCACAAGCGGTGGAGCATGTGGTTTAATTCGATGCAACGCGAAGAACCTTACCACTTCTTGACATGGAGAT |
| ON152891 | xyn32ph-1 | TACGAAGGTAATTAGTGGCAGACGGGTGAGTAATGCATAGGAATCTACCTAGTAGTATGGGATAGCCACTAGAAATGGTGGGTAATACTGTATAATCCCTGCGGGGGAAAGATTTATCGCTATTAGATGAGCCTATGTTAGATTAGCTAGTTGGTAGGGTAATGGCCTACCAAGGCAGTGATCTATAGCTGGTCTGAGAGGATGATCAGCCACACTGGAACTGAGATACGGTCCAGACTCCTACGGGAGGCAGCAGTGGGGAATATTGGACAATGGGCGCAAGCCTGATCCAGCTATGCCGCGTGAGTGAGGAAGGCCTTAGGGTTGTAAAACTCTTTCAGTAGGGAAGATAATGACGGTACCTACAGAAGAAGTCCCGGCAAACTCCGTGCCAGCAGCCGCGGTAATACGGAGGGGGCAAGCGTTGTTCGGAATTATTGGGCGTAAAGGGCATGTAGGCGGTTCGGTAAGTTAAAGGTGAAATGCCAGGGCTTAACCCTGGAGCTGCTTTTAATACTGCCAGACTAGAGACCGGGAGAGGATAGCGGAATTCCTAGTGTAGAGGTGAAATTCGTAGATATTAGGAGGAACACCAGTGGCGAAGGCGGCTATCTGGTCCGGTACTGACGCTGAGGTGCGAAAGCGTGGGGAGCAAACAGGATTAGATACCCTGGTAGTCCACGCTGTAAACGATGAGTGCTGAATGTGGGGATATTTTAT |
| ON152892 | xyn10ma-1 | CGGGGGAAAGATTTATCGCTATTAGATGAGCCTATGTCAGATTAGCTAGTTGGTGGGGTAATGGCCTACCAAGGCGGTGATCTGTAGCTGGTCTGAGAGGATGATCAGCCACACTGGAACTGAGACACGGTCCAGACTCCTACGGGAGGCAGCAGTGGGGAATATTGGACAATGGGCGCAAGCCTGATCCAGCTATGCCGCGTGAGTGAGGAAGGCCTTAGGGTTGTAAAACTCTTTCAGTAGGGAAGATAATGACGGTACCTACAGAAGAAGTCCCGGCAAACTCCGTGCCAGCAGCCGCGGTAATACGGAGGGGGCAAGCGTTGTTCGGAATTATTGGGCGTAAAGGGCATGTAGGCGGTTTGGTAAGTTAAAGGTGAAATACCAGGGCTTAACCCTGGGGCTGCTTTTAATACTGCAGGACTAGAGTCCGGAAGAGGATAGCGGAATTCCTAGTGTAGAGGTGAAATTCGTAGATATTAGGAGGAACACCAGTGGCGAAGGCGGCTGTCTGGTCCGGTACTGACGCTGAGGTGCGAAAGCGTGGGGAGCAAACAGGATTAGATACCCTGGTAGTCCACGCTGTAAACGATGAGTGCTGAATGTGGGGGCTTTTGCCTCTGTGTTGTAGCTAACGCGTTAAGCACTCCGCCTGGGGACTACGGTCGCAAGACTAAAACTCAAAGGAATTGACGGGGACCCGCACAAGCGGTGGAGCAT |
| ON152893 | 55-36b-bovis-2 | AATTAGTGGCAGACGGGTGAGTAATGCATAGGGACCTACCTAGTAGTATAGGATAGCCGCTAGAAGTGGTGGGTAATACTGTATAATCCCTGCGGGGGAAAGATTTATCGCTACATGATGAGCCTATGTTAGATTAGCTAGTTGGTGGGGTAATGGCCTACCAAGGCAGTGATCTATAGCTGGTCTGAGAGGATGATCAGCCACACTGGAACTGAGGCACGGTCCAGACTCCTACGGGAGGCAGCAGTGGGGAATATTGGACAATGGGCGCAAGCCTGATCCAGCTATGCCGCGTGAGTGAGGAAGGCCTTAGGGTTGTAAAACTCTTTCAGTGGGGAAGATAATGACGGTACCCACAGAAGAAGTCCCGGCAAACTCCGTGCCAGCAGCCGCGGTAATACGGAGGGGGCAAGCGTTGTTCGGAGTTATTGGGCGTAAAGGGCATGTAGGTGGTTTGGTTAGTTAAAGGTGAAATGCCAGGGCTTAACCCTGGAGCTGCTTTTAATACTGCCAGACTGGAGTCCCGGGGAGA |
| ON152894 | xyn113cr-1 | CGGGGGAAAGATTTATCGCTACTAGATGAGCCTATGTTAGATTAGCTAGTTGGTGGGGTAATGGCCTACCAAGGCGGTGATCTATAGCTGGTTTGAGAGGACGATCAGCCACACTGGAACTGAGATACGGTCCAGACTCCTACGGGAGGCAGCAGTGGGGAATATTGGACAATGGGCGCAAGCCTGATCCAGCCATGTCGCGTGAGTGAAGAAGGCCTTAGGGTTGTAAAACTCTTTCAG |
| ON152895 | cattle-1 | GTATTTTGACTATACAAAGGTATTGTAATAAGACTTTAATTGAGTGCTAAGAGAATGGATTTTCAAAAAAATTCTTTTTTAAGTTTAAAAATTAAAGTTATTTTTATTTGTGAAGAAACAATAATAAAAATTAAAGACAAGAAGACCCTATGAATTTTTATAAACTTTAATATTTAATTAAATATTAAAGTTTATTTAATTGGGGCGATTGAGAAAGATAAAAAACTTTTTTTTATTTAA |
| ON152896 | xyn10ma-1groel | GTCTGAAGATGAGATTGCACAGGTTGCTACTATATCTGCCAACGGAGATAAAAACATAGGTGGCAAGATAGCACAGTGCGTAAGGGAAGTTGGCAAAGACGGGGTAATAACGGTTGAGGAAAGCAAGGGGTTCAAGGACCTTGAGGTCGAAAGGACTGACGGTATGCAGTTTGACCGCGGATATCTCTCCCCTTACTTCGTCACCAACGCTGAGAAGATGCTGGTGGAGTTCGAGAATCCGTACATATTCCTAACTGAAAAGAAAATCAATCTAGTACAAAGCATACTGCCAGTACTAGAAAACGTCGCTAGGTCAGGAAGGCCATTGCTGATCATCGCGGAAGACGTAGAGGGTGAAGCATTAAGTACACTGGTGCTTAACAAGCTTCGCGGAGGCTTGCAGGTTGCAGCTGTCAAGGCACCTGGTTTTGGCGACAGAAGGAAAGACATGCTCGGCGATATTGCTGTAATAGCCGGTGCGAAATACGTGGTAAACGACGAGCTCGCAGTAAAGGTTGAGGACATCACTCTGGATGACCTCGGTACTGCTAAGACTGTGCGCATAACTAAGGATACAACCACAATCATAGGAAGCGTCGACAGCAACGCTGACAGCATTACCAGCAGAATTAGCCAGATTAAGTCTCAGATTGAAGTTTCTTCTTCTGACTACGACAAGGAAAAACTGAAGGAGAGGCTGGCGAAGCTCTCAGGTGGGGTTGCCGTACTTAAGGTTGGTGGCTCTAGTGAGGTTGAAGTCAAGGAGCGTAAGGATAGGGTTGAAGATGCTTTGCACGCAACTAGGGCTGCGG |
| ON016525 | Hedge-1 | TTTAATTGAGTGCTAAAAGAATGGAGTTTCAAAAAAAGTCTATCTTGAATTCAAAAATTGAATTTATTTTTATTTGTGAAGAAACAATAATTAAAATTAAGGACAAGAAGACCCTAAGAATTTTTAATTTTGTATATATAAATATATATATATACAAAATTTTAATTGGGGCGATTAAAAAATATAGTTAACTTTTAATTTTAAAAAATGATCCATTATTAGTGATTTAATGTAAAAAATACTCTAGGGATAACAGCGTAATAA |
| ON016526 | Hedge-2 | GTATTTTGACTATACAAAGGTATTGTAATAAGACTTTAATTGAGTGCTAAAAGAATGGAGTTTCAAAAAAAGTCTATCTTGAATTCAAAAATTGAATTTATTTTTATTTGTGAAGAAACAATAATTAAAATTAAGGACAAGAAGACCCTAAGAATTTTTAATTTTGTATATATATATATTTATATATACAAAATTTTAATTGGGGCGATTAAAAAATATAGTTAACTTTTAATTTAAAAAAT |
| ON016527 | Hedge-3 | GTATTTTGACTATACAAAGGTATTGTAATAAGACTTTAATTGAGTGCTAAAAGAATGGAGTTTCAAAAAAAGTCTATCTTGAATTCAAAAATTGAATTTATTTTTATTTGTGAAGAAACAATAATTAAAATTAAGGACAAGAAGACCCTAAGAATTTTTAATTTTGTATATATATATTTATATATACAAAATTTTAATTGGGGCGATTAAAAAATATAGTTAACTTTTAATTTAAAAAATG |
| ON016528 | Hedge-4 | GTATTTTGACTATACAAAGGTATTGTAATAAGACTTTAATTGAGTGCTAAAAGAATGGAGTTTCAAAAAAAGGCTATCTTGAATTCAAAAATTGAATTTATTTTTATTTGTGAAGAAACAATAATTAAAATTAAGGACAAGAAGACCCTAAGAATTTTTAATTTTGTATATATATATATTTATATATACAAAATTTTAATTGGGGCGATTAAAAAATATAGTTAACTTTTAATTTAAAAAATGATCC |
| ON016529 | Hedgehog | TATAAACTAAAACTGACCTATAATCACCTAAGGGCATATAAATAAGTTATCTAGTTATAAACTTTGGTTGGGGTGACCTCGGAGAACAAAAAAACCTCCGAATGATTCTAATCTTAGACACACAAGTCAAAGCATGATCATTTATTGACCCAGTAAATTTTACTGATCAACGGAACAAGTTACCCTAGGAATACCAGCGCAATCAA |
