# Supplementary figures and images for: Co-existence of Multiple Anaplasma Species and Variants in Ticks Feeding on Hedgehogs or Cattle Poses Potential Threats of Anaplasmosis to Humans and Livestock in Eastern China
Source: Front Microbiol. 2022 Jun 10;13:913650. doi: 10.3389/fmicb.2022.913650 (PMC9226643; doi:10.3389/fmicb.2022.913650)

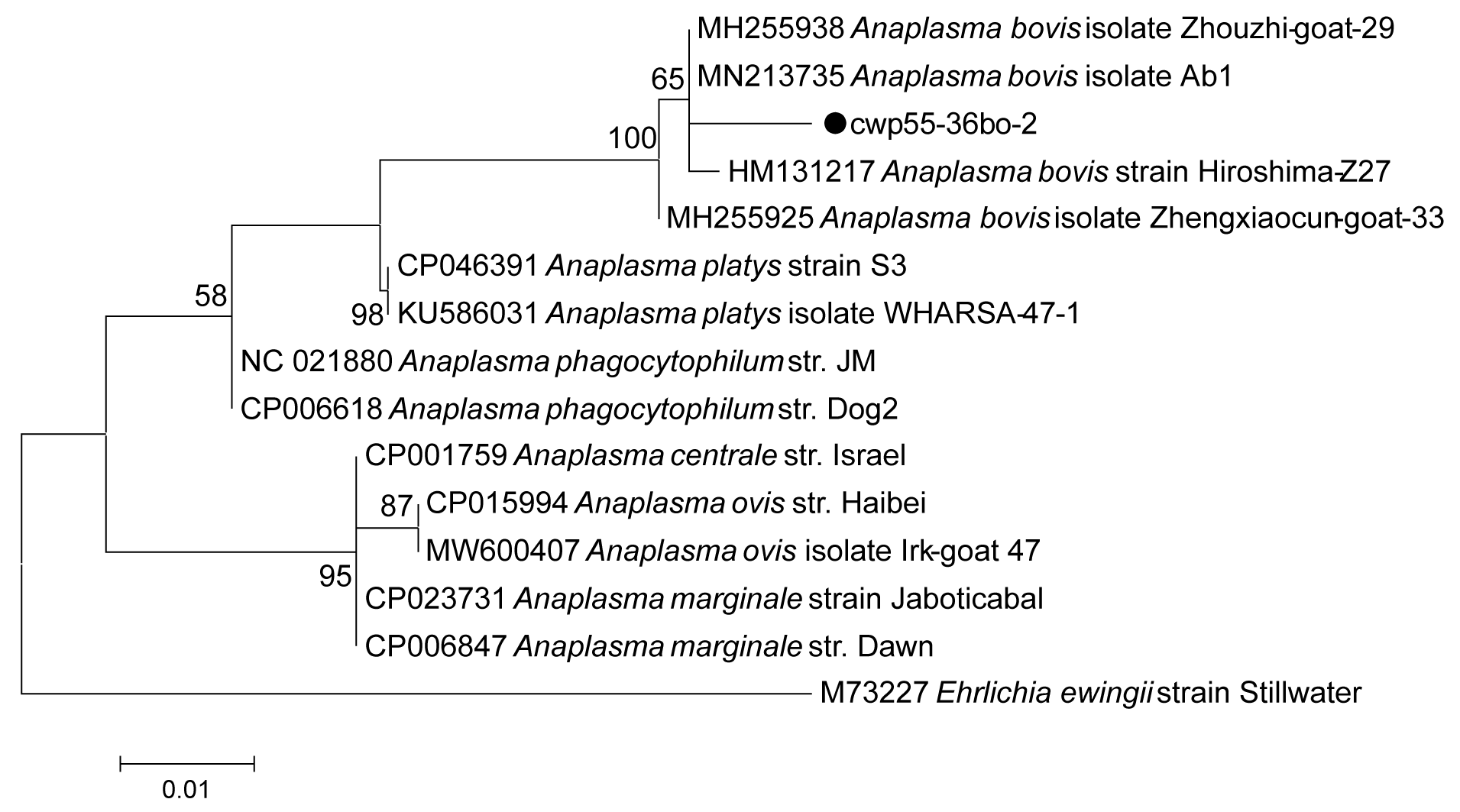

Supplement: Supplementary file 2 [file Image_1.TIF]

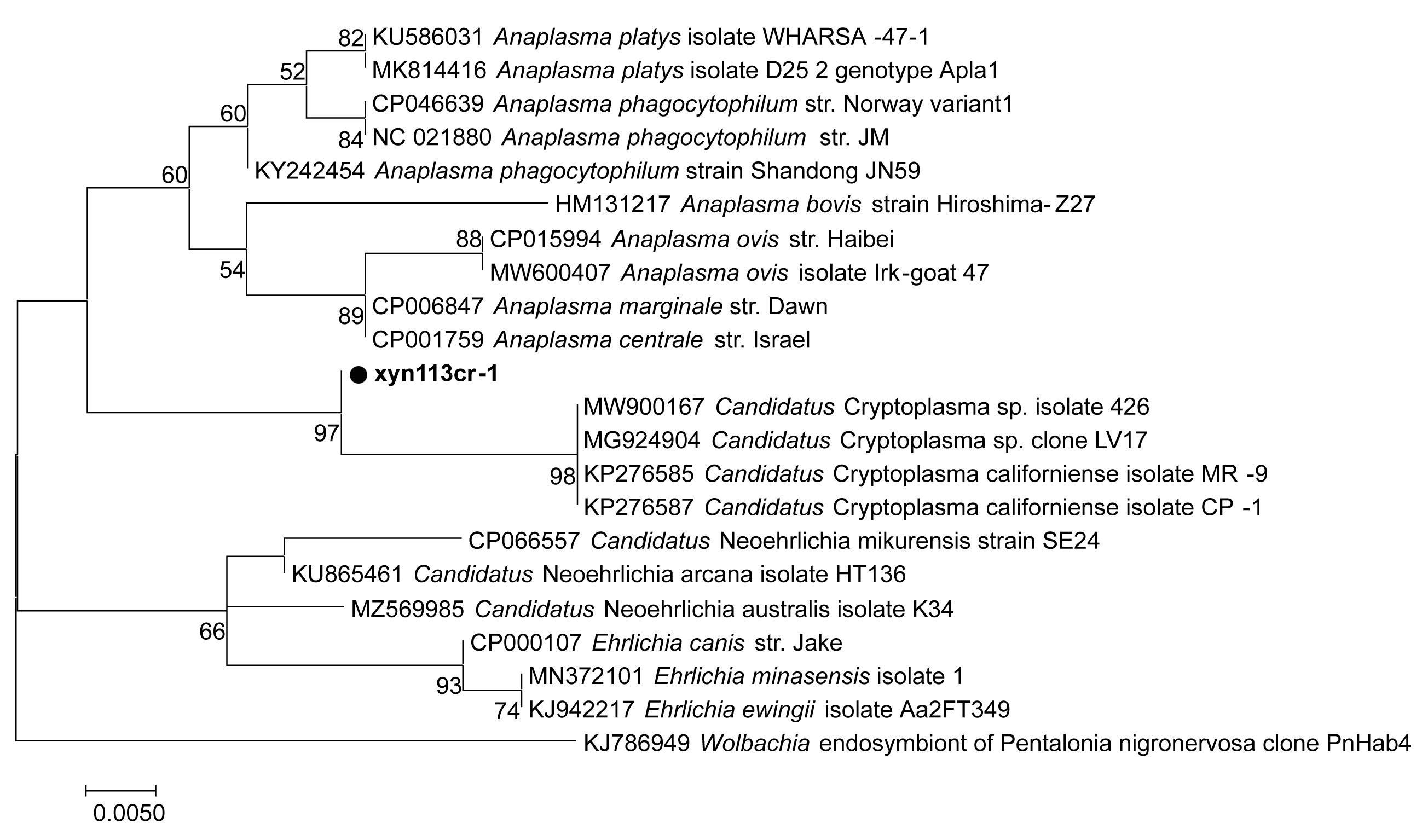

Supplement: Supplementary file 3 [file Image_2.TIF]
